# Study of local and non-local post-midnight equatorial spread-F generation based on long-term AMISR-14 observations

**DOI:** 10.1186/s40623-025-02319-1

**Published:** 2025-11-27

**Authors:** Alexander A. Massoud, Fabiano S. Rodrigues, Jonas Sousasantos, Karim M. Kuyeng, Danny E. Scipión, Carlos Padin

**Affiliations:** 1 The University of Texas at Dallas, William B. Hanson Center for Space Sciences, Richardson, TX 75080 USA; 2 Instituto Geofísico del Perú, Radio Observatorio de Jicamarca, Lima, Perú; 3 Universidad Ana G. Méndez, San Juan, PR USA

**Keywords:** Ionosphere, Post-midnight, Equatorial spread F, Geomagnetic storm, Phased array, Electronic beam-steering

## Abstract

We present results of a study of post-midnight equatorial spread F (ESF) events over the Jicamarca Radio Observatory (JRO) that examined unambiguous radar measurements of event origin in the American sector. Our analysis considers variations in post-midnight ESF generation due to changing seasonal, solar, and geomagnetic conditions. We analyzed 396 nights of observations made with the 14-panel version of the Advanced Modular Incoherent Scatter Radar (AMISR-14) between July 2021 and August 2023. We leveraged the 10-beam AMISR-14 mode, which effectively measures ~ 400 km zonally of the equatorial F-region ionosphere, to identify and classify post-midnight ESF as either local (i.e., generated within the instrument field of view) or non-local (i.e., generated outside the instrument field of view). Our results for the occurrence rates of post-midnight ESF exhibit a strong seasonal dependence, with maximum values in June solstice and minimum values for equinoxes. The results also show the post-midnight ESF occurrence rates are anticorrelated to the solar flux conditions. As for geomagnetic activity, the results indicate that occurrence rates decrease considerably under geomagnetically quiet conditions. The combination of these seasonal, solar flux, and geomagnetic activity influences suggests the weakened downward plasma drifts late at night during June solstice conditions can be reversed to upward drifts by contributions from disturbance drifts. In the case of upward drifts caused by geomagnetic disturbances, the reversed upward post-midnight drifts may then contribute to conditions favoring ESF development provided that a prompt penetration or disturbance dynamo electric field with appropriate polarity, even from modest geomagnetic activity, is present. In support of this proposed post-midnight ESF generation mechanism, we also present and discuss simultaneous AMISR-14 and collocated incoherent scatter radar measurements of a June solstice 2023 event. Perhaps most importantly, our results show the occurrence rates of local and non-local post-midnight ESF as observed with AMISR-14 are nearly identical. That is, local events were observed effectively as often as non-local events, and vice versa, under all seasonal, solar, and geomagnetic conditions. Therefore, data-driven forecasting approaches relying exclusively on local (i.e., “overhead”) measurements of ionospheric/thermospheric conditions may not always be well-suited to reproducing the observed ESF phenomenology.

**Graphical Abstract:**

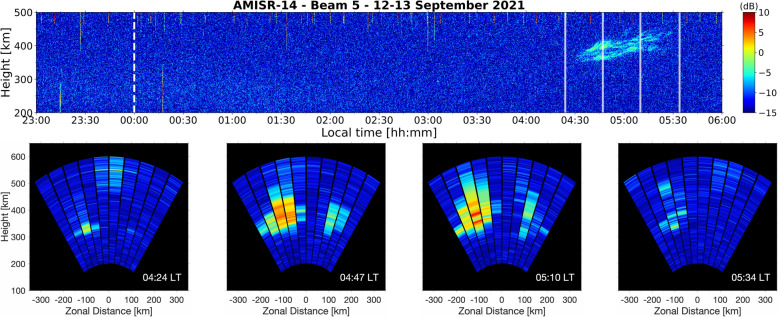

## Key Points

- We analyzed ~2 years of two-dimensional radar measurements to determine the climatology of post-midnight ESF generated locally (i.e., within the radar field of view) and non-locally.
- We found nearly the same (50/50) occurrence rates for post-midnight ESF events that developed locally and non-locally, independent of season and solar flux conditions.
- Collocated ESF and drift observations show the unequivocal case of a post-midnight ESF event generated under conditions of abnormal vertical plasma drifts.
- The observations also show that even moderate geomagnetic activity can contribute to the generation of post-midnight ESF during June solstice.
- The observations support the hypothesis that post-midnight ESF is more likely to occur under certain conditions of weak post-midnight drifts with contributions from disturbance electric fields.

## Introduction

Equatorial spread F (ESF) is the common term used to describe distinct signatures in a variety of space- and ground-based measurements that are the result of irregularities in the ionospheric F-region plasma density (Booker and Wells 1938; Hanson and Sanatani 1971; Woodman and LaHoz, 1976; Hysell 2000).

To explain the mechanism by which these irregularities develop into irregularities associated with ESF, the Generalized Rayleigh–Taylor (GRT) instability is often invoked (Burke et al. 1980; Kelley et al. 1981; Sultan 1996). The strong ionospheric upward drifts during the prereversal enhancement (PRE) of the zonal electric field (Rishbeth 1971; Heelis et al. 1974; Farley et al. 1986; Haerendel and Eccles 1992) at magnetic equatorial latitudes around local sunset are often considered the main factor controlling the development of GRT instabilities and subsequent ESF irregularities (Basu et al. 1996; Fejer et al. 1999). ESF is observed in the post-sunset sector (i.e., before local midnight) with high occurrence rates relative to other times (Abdu et al. 1998; Hysell and Burcham 2002; Smith et al. 2016; Zhan et al. 2018).

Despite the well-understood relationship between the PRE around sunset and the onset of ESF, atypical ESF events have still been reported for seasons and local times when conditions are not expected to be conducive to their development. For instance, numerous previous studies have described ESF events measured after local midnight, which are referred to as post-midnight ESF (e.g., Patra et al. 2009; Li et al. 2011; Yizengaw et al. 2013; Zhan et al. 2018). The development of such events, however, is yet to be fully understood.

Post-midnight ESF occurrence rates consistently reach maximum values during low solar flux solstices, especially the June solstice (Heelis et al. 2010; Smith et al. 2016; Zhan et al. 2018). Conventional assessments of the GRT instability linear growth rate suggest ESF should be effectively suppressed under these conditions. More recent studies of post-midnight ESF have suggested these events could have been caused by abnormal upward plasma drifts late at night (Stoneback et al. 2011; Zhan et al. 2018; Otsuka 2018; Krall et al. 2021; Chou et al. 2024). However, progress has been limited in part by difficulties in determining when and where post-midnight ESF originates and the limited availability of equatorial vertical drift measurements.

Efforts have been dedicated to identifying the origin of post-midnight ESF detected by radars. Collocated instruments such as Digisondes have been used to show an apparent uplift of the bottomside F-region prior to the onset of radar echoes associated with post-midnight ESF (Nicolls, 2006; Zhan et al. 2018; Rodrigues et al. 2019). Rodrigues et al. (2019) also performed spectral analysis of post-midnight F-region echoes and used echo spectral widths to infer the “age” (i.e., level of turbulence) of post-midnight ESF.

Furthermore, the electronic beam-steering capability of the Equatorial Atmosphere Radar (EAR) in Indonesia has enabled observations that capture the temporal and spatial development of post-midnight field-aligned irregularities (Otsuka et al. 2009; Yokoyama et al. 2011; Ajith et al. 2015, 2024). The use of this type of observational capability at magnetic equatorial latitudes would enhance understanding of the origin of post-midnight ESF over a certain location.

Since 2014, an electronic beam-steering capable radar has been deployed at the Jicamarca Radio Observatory (JRO, 11.95ºS geographic latitude, 76.97ºW geographic longitude, ~ 1ºS dip latitude). The radar is a smaller, 14-panel version of the Advanced Modular Incoherent Scatter Radar (AMISR) system and is referred to as AMISR-14. Rodrigues et al. (2015) showed the capability of AMISR-14 to measure Bragg scattering of ESF irregularities in look directions other than directly overhead. We emphasize that AMISR-14 can directly measure echo-causing irregularities associated with ESF in the magnetic equator over a wide zonal (i.e., ~ 400 km at F-region heights) field of view (FOV). However, technical issues with the system prevented its continuous operation until after repairs were completed in 2021.

Semi-routine (~ 200 days per year) observations of ESF with AMISR-14 began in July 2021 and have continued as part of regular observation campaigns at the JRO. Rodrigues et al. (2023) reported on the mode used for two-dimensional (2D) observations of ESF with AMISR-14. Massoud et al. (2024) reported on the occurrence rates of ultra-high frequency (UHF) echoes observed by AMISR-14 and how these rates responded to variations in season and solar flux conditions.

Here, we explore the new 2D AMISR-14 observations to advance our understanding of post-midnight ESF. More specifically, we use approximately two years of east–west “scans” of the ionospheric F-region made with AMISR-14 to determine how often post-midnight ESF events observed over Jicamarca developed locally, that is, within a few 100 s of km of the site. In addition, we compare 2D AMISR-14 “scans” which captured a post-midnight ESF event in June solstice 2023 to vertical plasma drift measurements made by a new collocated incoherent scatter radar (ISR) experiment. The simultaneous observations from both experiments allow us to describe the ionospheric dynamics under which post-midnight ESF above Jicamarca is observed.

This report is presented as follows: Sects. 2 and 3 provide an overview of AMISR-14, a description of its most relevant features, and example results from our analyses of post-midnight ESF events. In Sect. 4, we present results of our analyses of long-term observations of post-midnight ESF events made by AMISR-14. Emphasis is given to the effects of different geophysical factors (e.g., geomagnetic activity, solar flux, and season) on the occurrence of ESF events that develop near Jicamarca. In addition, results from AMISR-14 and a collocated ISR experiment are presented for a case study that provides new experimental evidence of the relation between abnormal drifts and post-midnight ESF. Finally, in Sect. 5, we summarize our main findings.

## Instrumentation and observations

The results presented in this study are obtained using measurements of UHF echoes made with the 14-panel version of the AMISR system—AMISR-14 (Rodrigues et al. 2015, 2023). For a detailed description of the AMISR system, see Valentic et al. (2013). UHF echoes observed with AMISR-14 are well-associatedwell associated with the occurrence of ESF irregularities, as described in Massoud et al. (2024). Here, we provide relevant information about AMISR-14 and describe our analysis of ESF events measured with the radar system after local midnight.

### AMISR-14

AMISR-14 experiments have complemented and expanded JRO observational capabilities (Rodrigues et al. 2015, 2018, 2023; Sousasantos et al. 2023; Green et al. 2023; Massoud et al. 2024; Hedges et al. 2025). The novelty of AMISR-14 in comparison to previous radar experiments at the JRO is demonstrated by the following two features: (1) operation in the UHF band (445 MHz) and (2) an electronic beam-steering capability. Previous radar experiments at the JRO did not have these unique features and thus could not investigate science questions that AMISR-14 can address. We briefly discuss both features below before describing the main parameters of the radar mode used to make observations used in this study.

Radar observations of ESF at Jicamarca have been made predominantly at 50 MHz, in the very-high frequency (VHF) band. Coherent backscatter echoes measured at 50 MHz are the result of Bragg scattering of ionospheric irregularities with scale sizes of ~ 3 m. AMISR-14 instead measures Bragg scattering of equatorial irregularities with sub-meter scale sizes of ~ 0.34 m. Rodrigues et al. (2015) presented the first observations of ESF made by AMISR-14. More recently, Massoud et al. (2024) used routine (i.e., ~ 200 days per year) AMISR-14 observations made between August 2021 and February 2023 to produce the first climatology of ESF echoes at UHF.

### AMISR-14 observations

For this study, emphasis is placed on the capability of AMISR-14 to electronically steer its beam and “scan” the ionospheric F-region in multiple directions. “Scans” or observations were made in a radar mode referred to here as the F-region mode. The F-region mode alternates through 10 different pointing directions. The radar mode is the same used by Rodrigues et al. (2023) for 2D observations of ESF. The azimuth and elevation angles of the 10 F-region mode pointing directions are listed in Table 1. Azimuth describes the angle of the antenna beam direction, measured clockwise from geographic north. Elevation describes the angle of the antenna beam direction, measured upward from the horizontal plane.

**Table 1:** Azimuth and elevation angles for each AMISR-14 F-region mode pointing direction

Beam number	Azimuth (degrees)	Elevation (degrees)
1	− 95.2	59.5
2	− 96.5	65.8
3	− 97.7	73.8
4	− 99.50	78.3
5	− 108.4	86.6
6	102.5	85.1
7	93.2	80.4
8	90.00	74.00
9	90.00	66.2
10	88.9	61.2

Table 1 shows the 10 pointing directions oriented in the magnetic equatorial plane. The uneven spacing in elevation angle between consecutive pointing directions was selected to ensure perpendicularity to the Earth’s magnetic field at F-region heights. For each pointing direction, AMISR-14 operates as a monostatic radar system that measures F-region coherent backscatter echoes of field-aligned irregularities in the magnetic equatorial plane.

Of main relevance to our analyses of AMISR-14 F-region mode observations is the wide FOV the observations provide. Although the finite beamwidth reduces the spatial resolution of AMISR-14 observations compared to results from interferometric in-beam imaging (e.g., Hysell and Chau 2006; Harding and Milla 2013), the F-region mode observations measure ESF over significantly larger zonal distances.

**Fig. 1:**
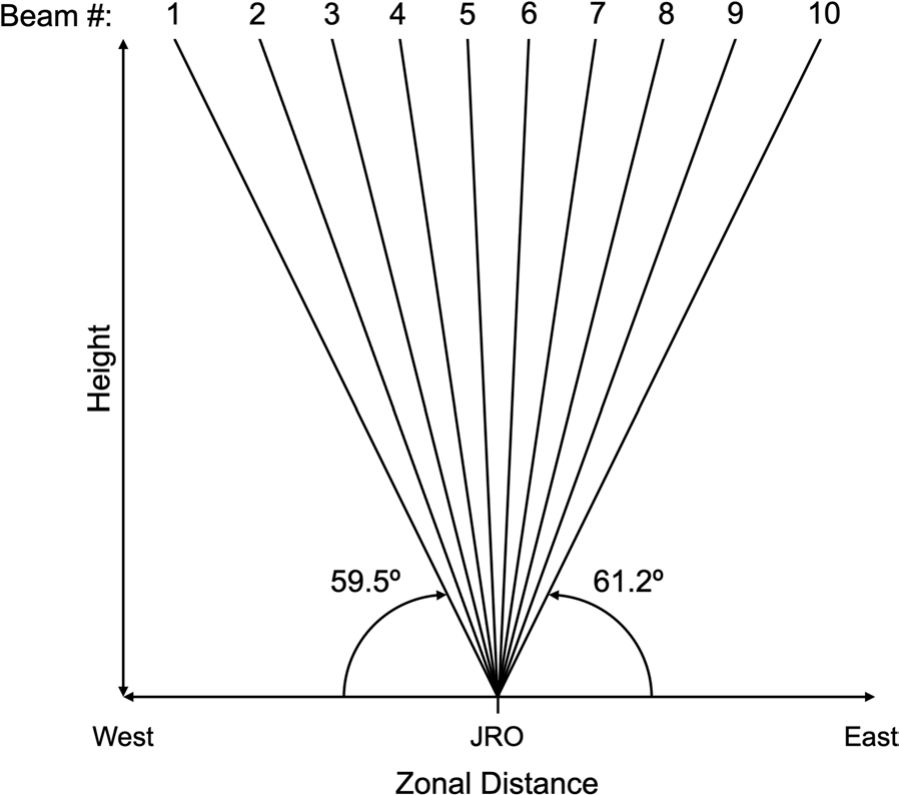
Sketch of the 10 pointing directions chosen for the antenna beam in the AMISR-14 F-region mode. All 10 pointing directions are in the magnetic equatorial (i.e., magnetic east–west) plane. Pointing directions are depicted relative to the Jicamarca Radio Observatory (JRO) in the figure. Labels for direction (i.e., east and west) indicate zonal distances relative to the JRO

Table 1 shows AMISR-14 observations obtained in the magnetic equatorial plane from an elevation of ~ 60º to the west of the JRO to ~ 60º to the east. Figure 1 provides a diagram visualizing the 10 pointing direction scans. Consideration of the values listed in Table 1 shows that, at 350 km in altitude, AMISR-14 “images” the ionosphere for ~ 200 km to the west and ~ 200 km to the east for a total zonal distance of ~ 400 km.

Table 2 lists experiment parameters for the AMISR-14 F-region mode. The time resolution of the scans is 20 s. Changes to the inter-pulse period (IPP), baud length, and number of pulses incoherently integrated were made to allow AMISR-14 to operate with other collocated VHF radar experiments and did not affect our analysis.

**Table 2:** AMISR-14 F-region mode experiment parameters

Parameter	16 Jul. 21 – 4 Sep. 22	5 Sep. 22 – 31 Oct. 22	1 Nov. 22–ongoing
Frequency	445 MHz	445 MHz	445 MHz
Bragg wavelength	0.34 m	0.34 m	0.34 m
Panel configuration*	7 (N/S) × 2 (E/W)	7 (N/S) × 2 (E/W)	7 (N/S) × 2 (E/W)
Antenna half power beam width (N/S and E/W)	1.4° (N/S) – 8.6° (E/W)	1.4° (N/S) – 8.6° (E/W)	1.4° (N/S) – 8.6° (E/W)
Nominal peak power	~ 185 kW	~ 185 kW	~ 185 kW
Number of beam positions	10	10	10
Pulses per beam position	16	16	10
Inter-pulse period (IPP)	937.5 km	1500 km	1500 km
Code length	28 bauds	28 bauds	28 bauds
Baud length	3.0 km	3.0 km	4.5 km
Sampling	1.5 km	1.5 km	1.5 km
Coherent integration	None	None	None
Incoherent integration	320 (2 s)	200 (2 s)	200 (2 s)

^*^ Panel configuration refers to how the 14 panels are placed. The configuration 7 (N/S) × 2 (E/W) refers to a rectangular configuration with 2 panels in the East–West direction and 7 panels in the North–South direction

The AMISR-14 observations used in this study were made during a portion of the ascending phase of solar cycle 25. We analyzed measurements made between 16 July 2021 (near the end of June solstice 2021) and 7 August 2023 (approximately the end of June solstice 2023). A total of 396 observations were available in this period. Thus, the observations capture ESF as solar flux conditions, geomagnetic conditions, and seasons changed.

## Analyses and examples of results

We performed analysis on ESF events that occurred over Jicamarca in the post-midnight sector. More specifically, we sought post-midnight echo events that would not appear as a continuation of pre-midnight ESF echoes in a range–time–intensity (RTI) map generated with traditional (i.e., single vertical pointing direction) monostatic radar. Therefore, we focused on events that started after midnight and appeared as an “isolated” echoing layer in traditional RTI maps.

Using AMISR-14 2D observations, we determined how often post-midnight events developed over Jicamarca locally. One important aspect of this investigation is that only local events can be adequately correlated with ionospheric/thermospheric conditions (e.g., vertical plasma drifts) measured over the observatory.

### Post-midnight ESF event in an RTI map

We visually inspected RTI maps generated from AMISR-14 measurements made between 16 July 2021 and 7 August 2023 to determine which observations could be used in our analysis of the post-midnight ESF events. Observations were removed from our analysis if more than ~ 30 min of measurements between local times (LT) of 22:00 LT and 06:00 LT were unavailable. The availability of measurements 2 h before midnight was considered in this step to ensure all events included for analysis that occurred soon after local midnight were “isolated” as described below. After this consideration, 396 observations were available for analysis.

We began by identifying echo events initially measured in the post-midnight sector with beam 5 and/or beam 6 of AMISR-14. Beams 5 and 6 are the two beams pointed closest to zenith (see Table 1). We chose to identify post-midnight events with beams 5 and 6 as opposed to the other eight off-zenith beams in order to emulate the close-to-zenith pointing direction used in traditional radar studies of post-midnight ESF at the JRO (Hysell and Burcham 2002; Smith et al. 2016; Zhan et al. 2018). The RTI maps for beams 5 and 6 most closely resemble RTI maps created with observations by VHF systems at the JRO (Massoud et al. 2024).

We then used RTI maps generated with beams 5 and 6 to identify post-midnight echo events that were isolated. An event was labeled as isolated if significant echoes were not measured for at least one hour before the onset of post-midnight echoes. Events that started in the post-sunset sector and continued until after midnight were not labeled as isolated. These events were removed from our analysis in an effort to focus on post-midnight echoes not associated with long-lasting pre-midnight ESF.

Finally, we considered isolated post-midnight echo events that displayed significant vertical development in beam 5 and/or beam 6. More specifically, an event had to extend for at least ~ 50 km in altitude to be included in our analysis. This criterion is used to remove events resembling bottom-type events (Woodman and La Hoz 1976; Hysell and Burcham 1998) from our analyses. Bottom-type echoing layers are caused by the wind-driven gradient drift instability (Kudeki and Bhattacharya, 1999; Hysell et al. 2004). We are interested in bottomside and topside ESF events associated with the GRT instability.

**Fig. 2:**
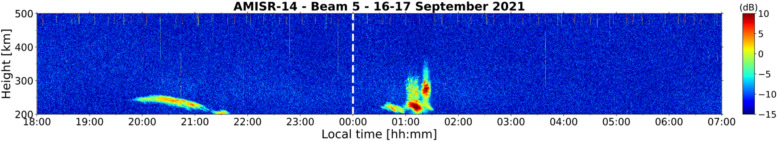
Range–time–intensity map of ultra-high frequency echoes measured by AMISR-14 beginning on 16 September 2021. Measurements correspond to one of the pointing directions closest to zenith (i.e., beam 5). The white dashed line marks local midnight

For illustration of our analysis, Fig. 2 shows an RTI map with an example post-midnight event that met our selection criteria. It shows observations made on 16–17 September 2021. The RTI map is for beam 5 and shows moderate-strength echoes from a thin scattering layer in the post-sunset sector on September 16. In the post-midnight sector, strong echoes from an echoing structure that reached ~300 km in height were measured. No significant coherent echoes were detected for ~3 h before the onset of post-midnight ESF. The RTI in Fig. 2 is analogous to one obtained with traditional radar experiments at the JRO.

The example in Fig. 2 also illustrates the type of observation where one can question whether the post-midnight ESF developed locally or to the west/east of the site. We reiterate that this question cannot be directly addressed by traditional radar experiments.

### Non-local post-midnight ESF event

AMISR-14 enables explicit measurement of ESF development when all 10 beams in the F-region mode are considered. As described in Sect. 2.2, AMISR-14 F-region mode observations “scan” the ionosphere at typical F-region heights over a zonal distance of ~ 400 km. The AMISR-14 observations made with the 10-beam F-region mode therefore allow us to distinguish between ESF that generated within the wide instrument FOV or entered the FOV already well-developed. To this end, we analyzed sequences of 2D (i.e., height versus zonal distance) images of UHF echoes.

We constructed 2D “snapshots” or “images” of the distribution of coherent backscatter echoes measured with the 10 F-region mode beams on nights with isolated post-midnight ESF. The images resemble the 2D plots, often referred to as fan sector plots or maps, generated with other radars that have an electronic beam-steering capability (e.g., Fukao et al. 2004; Ajith et al. 2015; Chen et al. 2017). Images with a temporal resolution of 20 s were combined in sequence to visualize the evolution of UHF echoes and associated ESF within the AMISR-14 FOV.

We used the sequences of 2D images to identify whether each isolated post-midnight ESF event was associated with echoing structures that developed within the AMISR-14 FOV (i.e., “fresh” or local) or developed in a longitude sector outside the FOV before they drifted in (i.e., “drifting-in” or non-local).

An event was labeled as local if vertically developed echoes were first measured in at least one of beams 2 through 9. First echoes had to be measured in beam 1, the beam pointed furthest to the west, or beam 10, the beam pointed furthest to the east, for an event to be labeled as non-local. Our analysis of the 2D images is similar to the methodology described by Ajith et al. (2015) for EAR measurements to describe the origin of coherent backscatter echoes caused by field-aligned irregularities in the low-latitude ionosphere.

**Fig. 3:**
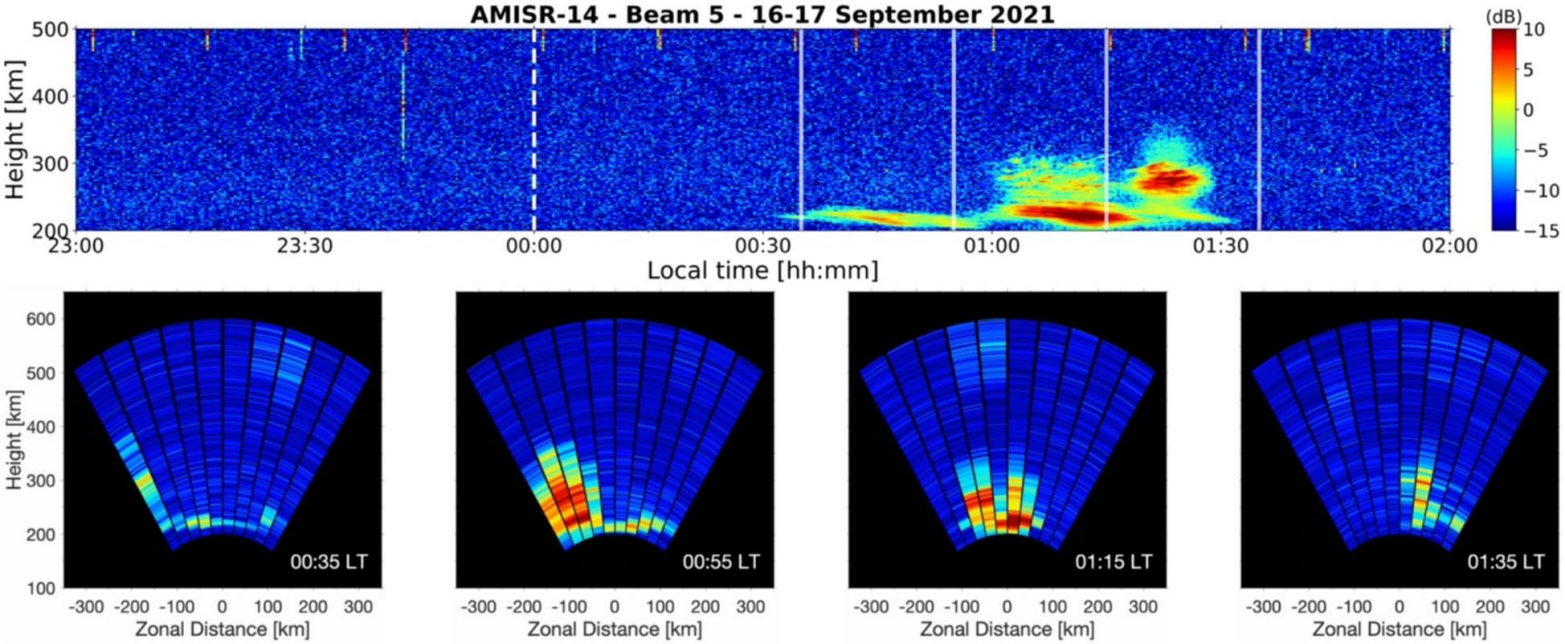
Range–time–intensity map of ultra-high frequency (UHF) echoes measured by AMISR-14 beginning on 16 September 2021 (top panel). Measurements are shown from one of the beams closest to zenith (beam 5). The white dashed line indicates local midnight. The transparent white solid lines mark the time of each “snapshot” in the bottom row. Two-dimensional images of the height versus zonal distance distribution of UHF echoes (bottom panels). The timestamp of each snapshot is indicated in the bottom right of each panel

Figure 3 shows an example of our analyses applied to the post-midnight event shown in Fig. 2. The top panel of Fig. 3 shows, again, the RTI map for beam 5 of the AMISR-14 observations. Figure 3, however, only shows measurements between 23:00 LT on September 16 and 02:00 LT on September 17. The bottom panels show a sequence of 2D images for a period between 00:35 LT and 01:35 LT. The arrangement of AMISR-14 beams in each image is the same as the sketch shown in Fig. 1, with beam 1 (the most westward beam) the furthest beam on the left and beam 10 (the most eastward beam) the furthest beam on the right. Negative zonal distances therefore correspond to observations to the west of the JRO, while positive zonal distances correspond to observations to the east.

The 2D images in the bottom row of Fig. 3 allow us to identify the UHF echoes associated with the isolated post-midnight ESF event as non-local. At 00:35 LT, moderate-strength UHF echoes that extended > 50 km in altitude were first measured in beam 1. Strong echoes were observed in sequence by the other western beams and then by the two beams (5 and 6) pointed closest to zenith. Finally, the UHF echoing structure produced weaker echoes in the eastern beams at 01:35 as it decayed vertically and zonally. It can be determined unambiguously from the sequence of 2D images that the event identified with the vertical beams originated non-locally.

Rodrigues et al. (2023) used similar sequences of 2D images made with F-region mode observations to describe the spatio-temporal dynamics (irregularity drift direction, spacing between structures, etc.) of different types of ESF measured on a single night. The present study uses the 2D observations to investigate the occurrence of isolated post-midnight ESF events and identify each event’s origin.

### Local post-midnight ESF event

**Fig. 4:**
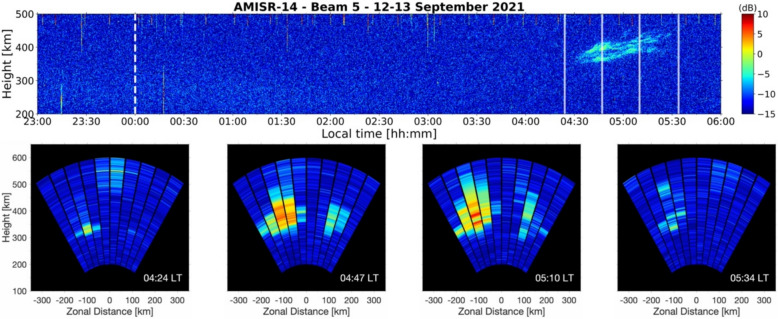
Beam 5 range–time–intensity map for AMISR-14 observations beginning on 12 September 2021 (top panel). The white dashed line indicates local midnight. The white transparent solid lines mark the time of each “snapshot” in the bottom row. Two-dimensional images of the observations with all 10 beams (bottom panels). “Snapshot” timestamps are indicated in the bottom right of each panel

For completeness, Fig. 4 now presents a case of isolated post-midnight ESF that was generated locally. The RTI map generated with beam 5 measurements beginning on 12 September 2021 is shown in the top panel of Fig. 4. Measurements are shown from 23:00 LT on 12 September to 06:00 LT on 13 September. The first ~ 5 h of observations shown do not display UHF echoes associated with ESF. After ~ 04:30 LT, isolated weak and diffuse echoes with a vertical development of ~ 50 km were measured.

The detection of weak echoes in the post-midnight sector has been interpreted in previous studies as a signature of F-region irregularities that formed far from the radar site long before the time of observation, which are referred to as “fossil” ESF (e.g., Sekar et al. 2007; Rodrigues et al. 2019). An analysis based solely on the RTI map in Fig. 4, which resembles observations with traditional radar experiments, could describe the isolated post-midnight ESF event as an example of “fossil” ESF. In such an evaluation, the event would be labeled as non-local.

The sequence of 2D images between 04:24 LT and 05:34 LT shown in the bottom panels of Fig. 4 reveals the isolated post-midnight ESF event generated locally. At 04:24 LT, moderate UHF echoes that extended ~ 50 km in altitude were first measured ~ 100 km to the west of the JRO in beam 3. Irregularities causing echoes to the west of the JRO grew vertically and horizontally and were measured in beam 5 at later times. The relatively weak echoes in beam 5 can be explained by the fact that the irregularities did not enter the main direction of the beam. Another region of irregularities produced weak vertically developed UHF echoes ~ 100 km to the east from ~ 04:45 LT to ~ 05:15 LT. By 05:34 LT, the echoing structure to the east had decayed, and only weak echoes associated with the echoing structure to the west were visible in the AMISR-14 FOV. The first 2D image in the sequence in Fig. 4 shows unambiguously that the event, despite expectations for “fossil” ESF, was in fact local.

Local ESF events typically show, initially, as thin scattering layers that develop vertically as time progresses. They also tend to move in the eastward direction. In some cases, however, like the example shown in Fig. 4, the ESF structure does not show a clear zonal motion. In very few cases, westward motion is observed. The non-local events tend to move in the eastward direction. In some cases, they develop vertically while moving zonally. In other cases, the maximum altitude of the ESF structures decreases as they traverse the radar FOV and, in some cases, they completely disappear (decay) before reaching the other side of the radar FOV.

## Results and discussion

We now present and discuss results of our analyses of approximately 2 years of 2D images of AMISR-14 observations with emphasis on determining the occurrence rates of local and non-local post-midnight events.

Table 3 summarizes the distribution of observations throughout the ~ 2-year period of analysis for this study. It shows the number of observations available for each season and each year between June solstice 2021 and June solstice 2023. The bounds of each season were set to ± 45 days from 21 March, June, September, and December, respectively. Please note that in order to include two events identified in measurements that began during the evening of 6 August in our analysis, the right bound of each June solstice was adjusted to be + 46 days from 21 June. Table 3 also indicates the number of observations made during geomagnetic quiet conditions (values in parentheses). In general, 40 or more observations are available each season. The exceptions are June solstice 2021 and June solstice 2022 when 18 and 24 observations were available, respectively.

**Table 3:** Number of observations analyzed and number of local/non-local isolated post-midnight equatorial spread F events organized by year and season. Values for geomagnetically quiet time are indicated in parentheses

	**2021**			**2022**			**2023**		
Season	No. of observations	No. of local events	No. of non-local events	No. of observations	No. of local events	No. of non-local events	No. of observations	No. of local events	No. of non-local events
Mar. equi	–	–	–	56 (20)	4 (1)	2 (0)	40 (11)	0 (0)	1 (0)
June sols	18 (11)	2 (1)	4 (2)	24 (14)	3 (1)	2 (1)	40 (14)	1 (0)	2 (0)
Sep. equi	43 (30)	5 (2)	3 (1)	65 (21)	3 (1)	3 (0)	–	–	–
Dec. sols	51 (27)	6 (3)	4 (3)	59 (24)	3 (2)	3 (2)	–	–	–

Before discussing our results, we must describe how we chose to distinguish between observations performed under geomagnetically disturbed and quiet conditions. The 3-h geomagnetic Planetary K (Kp) index values during and twelve hours before each observation had to be less than 3 for an observation to be considered quiet. More specifically, quiet observations were those for which the five Kp indices overlapping the observation period, 16:00 LT to 07:00 LT (21:00 UT to 12:00 UT), and the four previous Kp indices, 04:00 LT to 16:00 LT (09:00 UT to 21:00 UT), did not exceed 2.67. All 9 Kp indices were considered in order to identify isolated post-midnight ESF events potentially affected by geomagnetic activity. ESF is known to be affected by geomagnetic activity through penetration electric fields (Kikuchi et al. 1978; Senior and Blanc 1984), storm-time equatorward neutral winds, and/or disturbed dynamo electric fields (Blanc and Richmond 1980; Scherliess and Fejer 1997; Hysell and Burcham 2002; Balan et al. 2018).

Table 3 also shows, for each season, the results of our analyses of post-midnight event origin. It lists the number of local and non-local events identified in the 2D AMISR-14 observations. In the following sections, we will examine the results of our analyses in terms of occurrence rate and variation with solar flux and season.

### Local vs. non-local events: variations with solar flux

Here, we discuss changes in the occurrence rate of isolated post-midnight ESF events attributed to solar flux activity throughout the two years of AMISR-14 F-region mode observations considered for analysis.

Table 4 summarizes the observed occurrence rates of isolated post-midnight events according to year. Rates are computed as the number of events over the total number of observations for each analyzed period. We reiterate that observations were made during the ascending phase of solar cycle 25. The mean solar flux (F10.7) indices with standard deviation for the periods under which observations were made are indicated in Table 4. Mean solar flux conditions increased from 88.1 SFU (85.2 SFU) for the period of interest in 2021 to 165.4 SFU (165.7 SFU) in 2023 (1 SFU = 10^–22^ W m^−2^ Hz^−1^).

**Table 4:** Occurrence rate of local/non-local isolated post-midnight ESF events and average daily F10.7 index with standard deviation for each period of analysis organized by year. Rates are calculated using values in Table 3. Values in parentheses indicate values corresponding to geomagnetically quiet conditions

	**2021**			**2022**			**2023**		
	Occurrence rate of local events	Occurrence rate of non-local events	Average F10.7 (SFU)	Occurrence rate of local events	Occurrence rate of non-local events	Average F10.7 (SFU)	Occurrence rate of local events	Occurrence rate of non-local events	Average F10.7 (SFU)
Analyzed period	11.6% (8.8%)	9.8% (8.8%)	88.1 ± 13.4 (85.2 ± 11.5)	6.4% (6.3%)	5.0% (3.8%)	131.6 ± 27.5 (128.8 ± 24.9)	1.3% (0.0%)	3.8% (0.0%)	165.4 ± 23.7 (165.7 ± 16.5)

Table 4 shows that yearly isolated post-midnight ESF event occurrence rates in the seasons analyzed for 2021 were greater than those for 2022 and 2023. Both local and non-local events occurred at approximately half their respective rates in 2022 compared to 2021. In 2023, events occurred significantly less, with four events captured in 80 days of observations. All occurrence rates were reduced when only geomagnetically quiet observations were considered. We also point out that all events observed in 2023 were detected during a period classified as geomagnetically disturbed.

The decrease in the yearly isolated post-midnight ESF event occurrence rates is well associated with the increase in solar flux as the ascending phase of solar cycle 25 progressed from June solstice 2021 to June solstice 2023. The decrease is apparent for both geomagnetically quiet and disturbed conditions. This is in agreement with previous radar studies of ESF variations with changes in solar flux (Hysell and Burcham 2002; Smith et al. 2016; Zhan et al. 2018). For instance, Zhan et al. (2018) analyzed long-term VHF coherent backscatter radar measurements made by the JULIA mode of the Jicamarca radar and showed that the occurrence rate of post-midnight echoes decreases with solar flux in all seasons. However, their analyses focused exclusively on observations made during geomagnetically quiet conditions.

The described effect of solar flux in Table 4 matches expectations for changes with solar activity of ionospheric/thermospheric drivers of nighttime F-region dynamics. As solar flux increases, the magnitude of the downward drifts following the PRE becomes larger (Smith et al. 2016). Stronger downward drifts during higher solar flux conditions dampen ESF in the post-midnight sector (Fejer et al. 1999). The weak post-midnight drifts during low solar flux also allow disturbance upward drifts to more effectively create conditions favoring the development of post-midnight ESF (Scherliess and Fejer 1997). Thermospheric neutral winds may also have an effect on the stability of the equatorial F-region later in the night that varies with solar flux. Fang et al. (2016) and Zhan and Rodrigues (2018) used numerical simulations to show the destabilizing effect of converging equatorward winds. They suggested the midnight temperature maximum (MTM), which is linked to the convergence of winds in both hemispheres toward the magnetic equator, can drive midnight/post-midnight upward drifts, particularly when the post-PRE downward drifts are weakened under low solar flux conditions. In brief, ionospheric drifts and thermospheric winds during low solar flux conditions more readily facilitate the generation of post-midnight ESF compared to periods of high solar flux (Ajith et al. 2021).

We highlight one further finding from the results in Table 4: there is not a clear predominance of occurrence rate for either local or non-local events. This is perhaps the most important finding of our study. The results show, for the first time, that only half of the observed post-midnight events over Jicamarca originated locally. Therefore, only half the post-midnight events observed over Jicamarca could be directly associated with local background thermospheric and ionospheric conditions (e.g., vertical drifts) in any attempts to explain instability growth and irregularity development. This can explain why data-driven numerical efforts using local measurements failed to forecast late-night ESF events (e.g., Hysell et al. 2018).

Table 3 also shows trends in the occurrence statistics of isolated post-midnight ESF events that we attribute to changes in season. We discuss these trends in terms of seasonal occurrence rates in the following section.

### Local vs. non-local events: season-dependent occurrence rates

In this section, we address the extent to which the occurrence rate of isolated post-midnight ESF varies with season. Reported seasonal occurrence rates are computed as the number of events over the total number of observations in each season during either quiet or disturbed geomagnetic conditions.

Table 5 shows the seasonal occurrence rates of local and non-local post-midnight ESF events organized by year. Values for geomagnetically quiet conditions are listed in parentheses. The results in Table 5 show that occurrence rates of isolated post-midnight ESF events change with season.

**Table 5:** Occurrence rate of local/non-local isolated post-midnight ESF organized by year and season. Rates are calculated using values for the number of observations and number of events in Table 3. Rates for geomagnetically quiet observations are indicated in parentheses

	2021			2022			2023	
Season	Occurrence rate of local events	Occurrence rate of non-local events		Occurrence rate of local events	Occurrence rate of non-local events		Occurrence rate of local events	Occurrence rate of non-local events
Mar. equi	–	–		7.1% (5.0%)	3.6% (0.0%)		0.0% (0.0%)	2.5% (0.0%)
June sols	11.1% (9.1%)	22.2% (18.2%)		12.5% (7.1%)	8.3% (7.1%)		2.5% (0.0%)	5.0% (0.0%)
Sep. equi	11.6% (6.7%)	7.0% (3.3%)		4.6% (4.8%)	4.6% (0.0%)		–	–
Dec. sols	11.8% (11.1%)	7.8% (11.1%)		5.1% (8.3%)	5.1% (8.3%)		–	–

When observations made during any geomagnetic conditions are considered, the highest occurrence rates of events were found for June solstice (33.3%, 20.8%, and 7.5% for 2021, 2022, and 2023, respectively). When exclusively geomagnetic quiet events are considered, seasonal occurrence rates reached maximum values during June solstice in 2021 (27.3%) and December solstice in 2022 (16.6%). The occurrence rate in December 2022 is only slightly higher than the occurrence in June 2022 (14.2%). Additionally, we must point out that a reduced number of observations were available for analysis in June solstice 2021 and 2022 compared to other seasons. Seasonal occurrence rates for each year were lowest during equinoxes under all geomagnetic conditions, with occurrence rates for geomagnetically quiet time not exceeding ~10%.

The observed seasonal variation of ESF occurrence rates corresponds well with seasonal variations in nighttime vertical plasma drifts measured above Jicamarca (Scherliess and Fejer 1999). After the PRE peak, the vertical drifts are typically downward during the remainder of the night. These late nighttime downward drifts decrease the linear growth rate of the GRT instability (Sultan 1996; Basu 2002), dampening the evolution of electron density irregularities later at night. Strong downward drifts during nighttime thus significantly reduce the linear growth rate and can decrease the occurrence rates of post-midnight ESF. It has been shown that the magnitude of the downward drifts over the Peruvian region is larger during equinox than solstice (Smith et al. 2016). Additionally, it has been found that weak downward drifts follow the weakened or absent PRE in June solstice.

The variation in seasonal occurrence of isolated post-midnight ESF events also agrees well with previous studies that used different observational techniques. At Jicamarca, noticeable occurrence rates of ESF have been found in the post-midnight sector during solstices (e.g., Hysell and Burcham 2002; Zhan et al. 2018; Massoud et al. 2024).

Smith et al. (2016) also used coherent scatter echoes measured with the Jicamarca ISR to obtain post-midnight ESF occurrence rates. They found values as high as 70% during the December solstice. We point out that the large power-aperture product of the Jicamarca ISR might enable the detection of weak post-midnight ionospheric irregularities that would not be captured by AMISR-14 or other low-power (i.e., kW peak power) experiments, such as the JULIA radar configuration. In addition, post-midnight ESF in the December solstice often begins its development in the post-sunset sector before persisting through midnight (Zhan et al. 2018) and would be removed from our analysis of only isolated events.

Here, we highlight that the results in Table 5 do not indicate any significant seasonal variations for the origin of isolated post-midnight ESF. That is, the occurrence of local events was not significantly greater than the occurrence of non-local events, and vice versa, for any season. The greatest difference in a season between the occurrence rates of local and non-local events (11.1%) was recorded in June solstice 2021. However, only two more non-local events than local events were measured to produce this difference. For all seasons in the analyzed period, the difference in the number of both types of events did not exceed 2.

Our results show AMISR-14 2D images can be used to identify post-midnight ESF events that developed near the site. We also showed, for different conditions of solar flux and season, that approximately half the isolated post-midnight ESF events detected by AMISR-14 developed locally.

In the following section, we present a case study showing the equatorial vertical drift conditions under which a local isolated post-midnight ESF event developed.

### On vertical plasma drifts preceding local post-midnight ESF

As mentioned earlier, the analysis of 2D observations by AMISR-14 was motivated by the fact that thermospheric and/or ionospheric conditions measured at an observation site can only be adequately associated with ESF events that developed locally, that is, near that site. Using the ability of AMISR-14 to unambiguously identify ESF event origin (e.g., Rodrigues et al. 2023), we showed that half of the isolated post-midnight ESF captured in observations made between July 2021 and August 2023 developed locally.

Additionally, like previous studies, we found that post-midnight ESF was observed with higher occurrence rates during the June solstice and low solar flux conditions. This could be explained by the impact of disturbance upward drifts acting on the relatively weak background drifts observed in the post-midnight sector during these conditions. It has been suggested that these disturbance drifts could be created by geomagnetic disturbances (e.g., Fejer 2011) or by polarization electric fields created by sporadic E layers or medium-scale traveling ionospheric disturbances (MSTIDs) during geomagnetic quiet conditions (e.g., Yizengaw et al. 2013).

In this section, we examine an example of ionospheric vertical drift conditions preceding the development of a local post-midnight ESF event measured by AMISR-14 on 1 June 2023. This case study was possible due to collocated AMISR-14 and ISR measurements. While AMISR-14 was making the 2D observations of ESF, concurrent observations of vertical drifts were being made as part of tests of a new medium power (MP) ISR mode.

The MP ISR uses two solid-state transmitters with a combined peak power of ~200 kW. The new transmitters can be operated with substantially less power and associated costs than previous ISR experiments performed using MW transmitters. MP ISR enables more frequent ISR measurements of certain ionospheric state parameters than were previously possible.

Different versions of the mode have been implemented to optimize the use of the new transmitters. For the present case study, the experiment being tested used coded (Barker 3) 45-km-long pulses to make measurements of ionospheric drifts and coherent echoes at F-region heights. The coded long pulses allow ISR measurements around F-region peak heights despite the reduced power (e.g., Kuyeng et al. 2023).

**Fig. 5:**
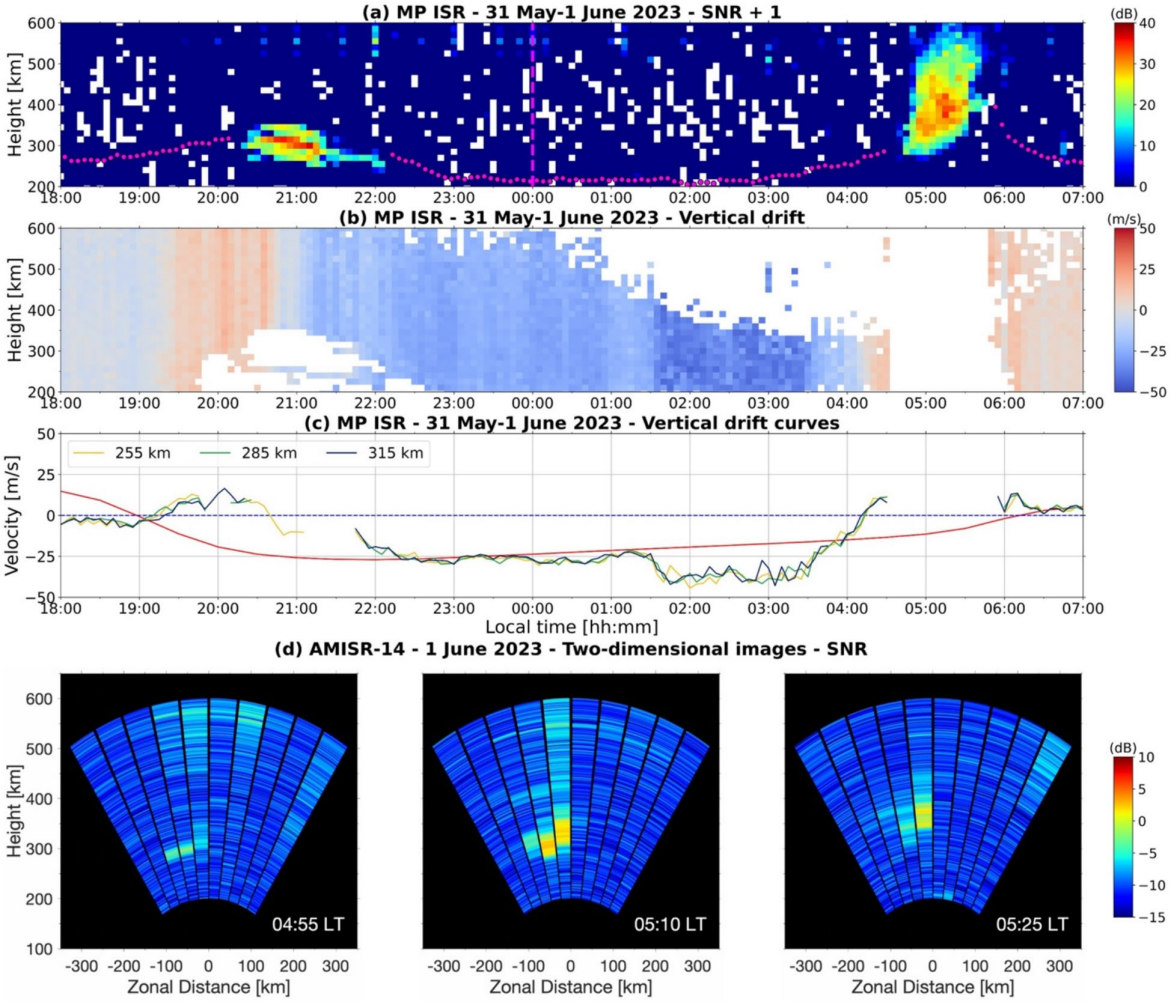
Range–time–intensity map of coherent echoes measured by the MP ISR between 18:00 LT on 31 May and 07:00 LT on 1 June 2023 (panel **a**). Local midnight is indicated by the dashed magenta line. The variation of the virtual height of the F-region—h’F (magenta circles) measured by a collocated ionosonde is also shown. Panels (**b**) and (**c**) show the vertical plasma drift measurements available for analysis and vertical drift curves for three heights: 255 km, 285 km, and 315 km. Panel (**c**) includes SF99 estimates of the vertical drifts (red curve). Sequence of AMISR-14 two-dimensional images generated from 10 beam measurements on the same night (panel **d**)

Figure 5 summarizes the post-midnight ESF event on 1 June 2023 and our analyses. Figure 5(a) shows the RTI map for MP ISR measurements. The measurements were made with a beam pointed nearly vertical, analogous to traditional radar measurements. The RTI map shows an ESF echoing layer confined to bottomside F-region heights in the post-sunset sector on 31 May 2023. The RTI map also shows an isolated post-midnight ESF event starting at around 04:45 LT on 1 June. Overlaid on the RTI map, we include the virtual height of the F-region base (h’F) as observed with a collocated ionosonde.

The MP ISR measurements alone do not enable a determination of whether this post-midnight event developed locally or drifted into the antenna beam from the east or west. The 2D images provided by AMISR-14, however, allow us to identify the event as local. A sequence of AMISR-14 images for an interval between 04:55 LT and 05:25 LT is shown in Fig. 5(d). The images show weak echoes starting around 04:55 LT. The echoes increase in strength and expand in altitude as shown in the images for 05:10 LT and 05:25 LT.

Figure 5(b) shows MP ISR vertical plasma drifts as a function of altitude and LT. It shows that drift measurements are not available where/when either ionospheric densities are very low or incoherent scatter echoes are obscured by much stronger coherent echoes of ESF irregularities. To further ensure that drift measurements were not affected by the combination of decreased densities and strong coherent echoes, we removed from our analysis drifts observed during and 10 min prior to the appearance of post-midnight ESF echoes in the RTI map (Panel a). The period of measurements not used in the analyses is represented by the white portion of Fig. 5(b) from 04:35 LT to 05:45 LT.

Figure 5(c) shows vertical plasma drifts at three different F-region heights: 255 km, 285 km, and 315 km. The curves show that the drifts do not vary much with height throughout the region of interest. Also included in Fig. 5(c) are estimates of the geomagnetic quiet time F-region vertical drifts provided by the commonly used Scherliess and Fejer (1999) empirical model (SF99).

The vertical drifts observed in the pre-midnight hours of May 31 show a weak PRE followed by the occurrence of what resembles a bottom-type layer. The SF99 model is representative of average conditions and does not show a distinguishable PRE peak. The PRE is known to have significant day-to-day variability (Liu 2020). This deviation between the mean drifts and SF99 output is discussed further in Sect. 4.4.

The MP ISR vertical drifts also show downward drifts with magnitude ~25 m/s after the PRE and throughout most of the night. This is in good agreement with previous studies and our hypothesis of nighttime downward drifts with substantial magnitudes during moderate and high solar flux conditions (see Sect. 4.1). The SF99 model shows drifts with magnitudes similar to the MP ISR mean drifts between ~ 22:00 LT and 01:30 LT.

Figure 5(c) shows that the magnitude of the downward drifts deviates significantly from the values provided by SF99 after ~ 01:30 LT. Additionally, it shows that the downward drifts begin to weaken around 03:30 LT and turn upward by ~ 04:30 LT, changing from ~ -35 m/s to ~ 10 m/s in approximately one hour. We emphasize that the negative-to-positive transition seen in the drift measurements occurred much quicker and earlier than what is shown by the SF99 model, suggesting a contribution from an external process. Perhaps more importantly, it is possible to identify that a significant change in the drift conditions including unexpected upward drifts preceded the appearance of the post-midnight plume in panel (a).

To provide more information about the conditions under which the post-midnight plume shown in panel (a) of Fig. 5 was generated, we also analyzed the local time variation of h’F measured by a collocated ionosonde.

The measurements show that the weakening in the downward drifts, which started around 03:00 LT, was followed by an increase in h’F. While h’F was initially at a height of about 210 km, it increased to approximately 286 km at 04:28 LT, prior to the development of the post-midnight ESF. The apparent uplift corresponds well with the weakening downward drifts starting around 03:30 LT (Nicolls et al. 2006) and matches the behavior of previous h’F observations prior to the June solstice post-midnight ESF (Zhan et al. 2018).

The development of ESF is commonly attributed to the GRT instability (Basu 2002). The upward drifts affect the GRT growth rate both directly and indirectly. They contribute directly to the linear growth rate through the $E/B$ term. They can also contribute indirectly by lifting the F-region to higher altitudes where the ion-neutral collision frequency ($\nu$_in_) decreases. As a result, the GRT linear instability growth rate will also have contributions from the gravitational term ($g/\nu$_in_). While the contribution of the gravitational term can be expected to be more effective during low solar flux conditions (Joshi et al. 2012), it can also contribute during the moderate-to-high solar flux conditions (F10.7 = 168.5 SFU) under which the observations of May 31–June 1, 2023, were made (Fig. 5).

Additionally, the increased h’F values observed before and after the detection of the plume confirm conditions favoring the generation of ESF in the post-midnight sector. Additionally, it confirms that the echoes were not the result of irregularities developing within a “fossil” large-scale ESF structure (e.g., Sekar et al. 2007). Finally, the h’F observations reinforce the finding from the 2D images in Fig. 5(d) that the post-midnight plume on 1 June 2023 was generated locally.

We must mention that while upward drifts are often cited as the most important parameter (Huang, 2008; Stolle et al. 2008; Kil et al. 2009), other factors (e.g., ionospheric scale height, neutral winds) can also contribute to the GRT linear growth rate and can make the magnitude of the upward drifts less critical for the development of ESF in the post-midnight sector. Additionally, non-local quantities can also contribute to the growth rate (Sultan 1996). For instance, the growth rate is expected to be increased further through the ${\Sigma }_{\text{P}}^{\text{F}}/({\Sigma }_{\text{P}}^{\text{E}}+{\Sigma }_{\text{P}}^{\text{F}})$ term of the flux tube-integrated linear growth rate expression for the GRT instability. ${\Sigma }_{\text{P}}^{\text{E}}$ and ${\Sigma }_{\text{P}}^{\text{F}}$ are the flux tube-integrated Pedersen conductivities at E- and F-region heights, respectively. Reduced off-equator E-region Pedersen conductivity late in the night could lower the threshold magnitude of the vertical plasma drifts required for the generation of ESF irregularities. This, perhaps, could explain the lack of well-developed ESF structures in the post-sunset sector of May 1, 2023 (Fig. 5) when the PRE drifts reached values that were similar to those seen prior to the post-midnight ESF event.

Most importantly, the observations show the unequivocal occurrence of abnormal vertical drifts contributing to conditions favoring the development of the observed post-midnight ESF over Jicamarca.

For completeness, in the following section we describe mechanisms through which the observed abnormal post-midnight drifts may have developed.

### On the observed post-midnight upward drifts

To provide context to the abnormal drifts observed prior to post-midnight ESF development, we examined the geomagnetic conditions and potential magnetosphere–ionosphere coupling processes that could have contributed to the observed behavior. For this purpose, the geomagnetic SYM-H, auroral electrojet (AE), and Kp indices and interplanetary solar wind magnetic field (IMF) z-component (Bz) parameter were used (King and Papitashvili 2005; Matzka et al. 2021).

**Fig. 6:**
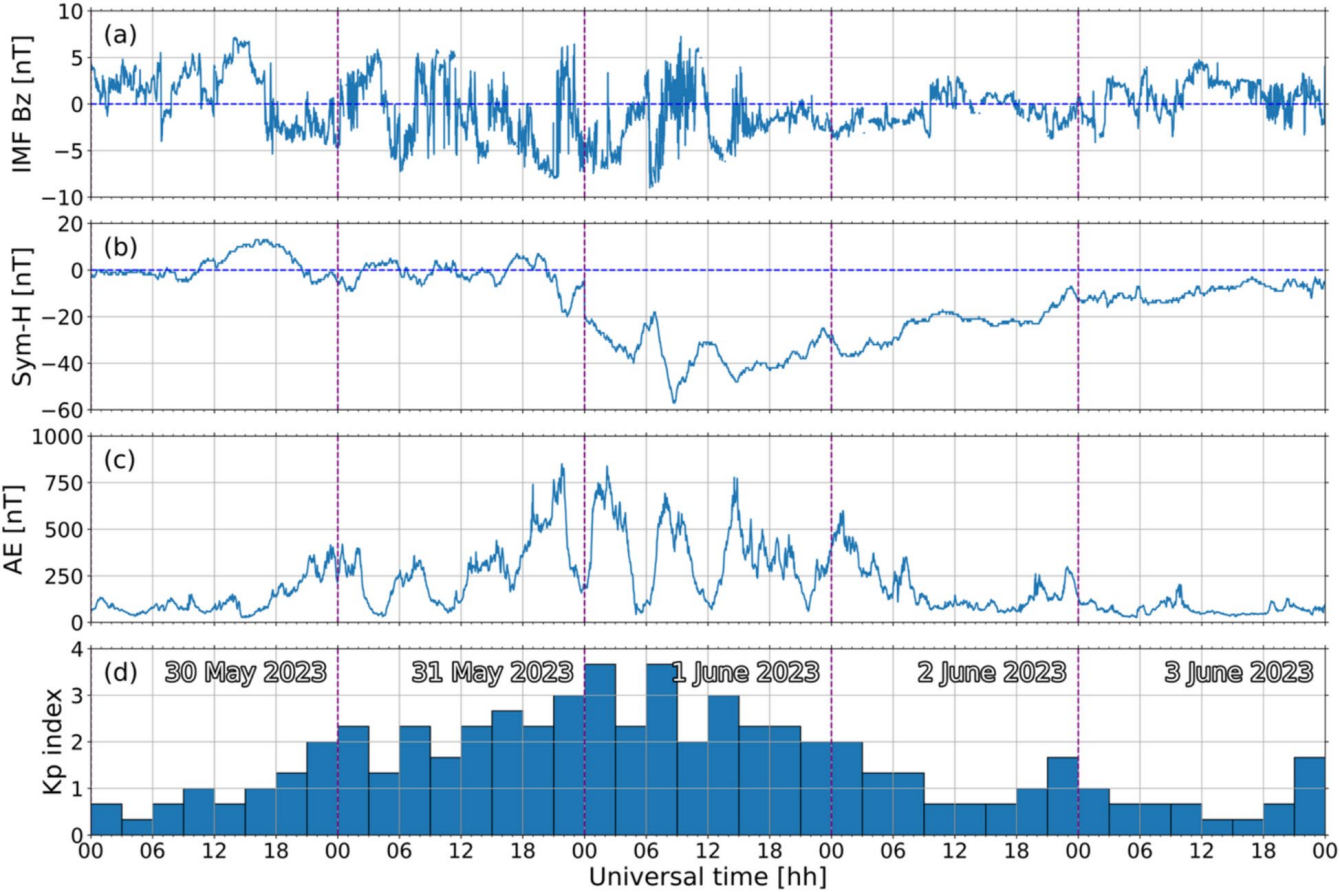
Interplanetary magnetic field z-component (panel **a**), SYM-H (panel **b**), auroral electrojet index (panel **c**), and Planetary K index (panel **d**) for 30 May to 3 June 2023. Please note the MP ISR observations shown in Fig. 5 covered the period from 18:00 LT (23:00 UT) on 31 May to 07:00 LT (12:00 UT) on 1 June 2023. Purple dashed lines indicate the start of a day. Dates are indicated as text in panel (**d**)

Figure 6 shows the variation of each index from 30 May to 3 June 2023 so their behavior before, during, and after the post-midnight event on 1 June 2023 can be inspected. The horizontal axis is presented in universal time (UT) because the indices are global. For the longitudinal sector of Jicamarca, LT $\approx$ UT—5.

Figure 6 reveals a geomagnetic storm which began around 20:00 UT on 31 May and ended roughly at 08:30 UT on 1 June 2023. Values in Fig. 6 for 30 May to the earlier hours of 31 May and 2 to 3 June 2023 represent the time preceding the onset and including the recovery phase, respectively. The start and end time of the storm are determined from the SYM-H values in panel (b). SYM-H reached a minimum value of approximately -55 nT. It can then be concluded that the storm was a moderate intensity one (Gonzalez et al. 1994; Collado-Villaverde et al. 2024).

Panel (c) of Fig. 5 shows deviations between the vertical plasma drift curves and the predicted values obtained with the SF99 empirical model. We emphasize and describe below the deviations for three periods during observations on the night of 31 May to 1 June 2023.

First, the mean vertical plasma drifts were more upward (i.e., positive or less negative) than SF99 output values between 19:15LT (00:15 UT) and 22:15 LT (03:15 UT). Later at around 01:30 LT (06:30 UT), the mean drifts were more downward (i.e., more negative) than values predicted by SF99. Finally, after ~ 03:30 LT (~ 08:30 UT), the mean drifts again turned more upward than those from SF99. The two upward drift deviations at ~ 19:15 LT and ~ 03:30 LT were both followed by the measurement of coherent echoes associated with ESF.

The first upward deviation in the mean drifts occurred in the early night – 19:15 LT (00:15 UT) – and coincided with the main phase of a moderate geomagnetic storm. The first IMF Bz southward turning during a time of more negative SYM-H occurred around 23:00 UT. A steep increase in the auroral activity as described by the AE index (~ 800 nT) was seen around 20:00 UT shortly followed by the geomagnetic activity as measured by the Kp reaching its largest value (3.67) around 00:00 UT. This synchronized development of auroral activity and upward drifts over the equatorial region suggests the contribution of eastward prompt penetration electric fields (PPEFs) (Fejer and Navarro 2022; Fejer et al 2024).

The second noticeable deviation occurred at ~ 01:30 LT (06:30 UT) and was predominantly downward. This deviation coincided with a second southward turning of the interplanetary Bz component which endured until ~ 08:00 UT. Again, PPEFs likely contributed to the deviation. However, since the electric fields penetrated to the equatorial region over Jicamarca after midnight LT, they had the opposite polarity (westward) of those in the early nighttime (Abdu 2012; Fejer et al. 2021). These westward PPEFs therefore contributed to downward equatorial vertical plasma drifts. Additionally, the downward deviation occurred ~ 6 h after Kp first reached its maximum value. Consequently, unlike the first upward deviation discussed above, the disturbance dynamo related to the first Kp peak had sufficient time to set up and influence the equatorial F-region dynamics. In the post-midnight sector, disturbance dynamo vertical drifts are upward (Navarro et al. 2019). Since the net mean drifts were more downward than the SF99 values and the geomagnetic storm was only moderate, we suggest the disturbance dynamo influence was superseded by the contribution from the PPEF related to the southward Bz turning.

The third most noticeable deviation occurred at ~ 03:30 LT (08:30 UT) and involved a rise of the mean drift. We identify a northward turning of the interplanetary Bz around ~ 07:50 UT (02:50 LT) that may have led to an overshielding electric field. This overshielding electric field contribution would have the same polarity as that from the disturbance dynamo discussed in the last paragraph, and their combined influence would lead to an enhanced upward drift.

We highlight that both upward deviations of the mean drifts preceded ESF echoes. Therefore, the vertical drift results in Fig. 5 show that during the June solstice, even under elevated solar flux (daily F10.7 = 168.5 SFU) conditions, the development of local post-midnight ESF events does not require strong geomagnetic disturbances. Instead, post-midnight ESF can develop locally if even mild geomagnetic activity contributions are present. The results reinforce the role of the upward drifts in fostering the development of post-midnight ESF.

## Conclusions

In this study, we analyzed 396 nights of AMISR-14 F-region mode observations made between July 2021 and August 2023 to identify isolated post-midnight ESF events (i.e., those with onset after 00:00 LT) and determine the occurrence rates of both locally and non-locally generated ESF events with changing geomagnetic, seasonal, and solar flux conditions. In order to do so, we used the AMISR-14 radar system in the 10-beam F-region mode to observe a wide (~ 400 km) zonal portion of the equatorial ionosphere.

Our long-term study indicates that the occurrence rates of post-midnight ESF generation are strongly dependent on season, with larger (lesser) occurrences in June solstice (equinox). We suggest this seasonal variation is related to the nighttime vertical plasma drifts over Jicamarca, since stronger late nighttime downward drifts during equinox are expected to dampen the growth of post-midnight ESF (Scherliess and Fejer 1999; Smith et al. 2016).

Our study also demonstrates the influence of solar flux conditions on the occurrence rates of isolated post-midnight ESF. As the yearly average solar flux increased, the occurrence rates decreased drastically. For example, an increase of ~ 40 SFU from 2021 to 2022 reduced yearly isolated post-midnight ESF occurrence rates by half. The variation in the occurrence rates attributed to solar flux is related to the expected behavior of vertical plasma drifts and meridional thermospheric neutral winds during low solar flux conditions (Fejer et al. 1999; Fesen 1996; Niranjan et al. 2003; Ajith et al. 2021).

As for the geomagnetic activity influence, all occurrence rates were lowered when only geomagnetically quiet measurements were considered. This result suggests the important role of geomagnetic activity in the development of post-midnight ESF locally over Jicamarca.

The case study using collocated AMISR-14 and MP ISR observations showed, unequivocally, the role of vertical drifts in the development of post-midnight ESF. In addition to affecting the GRT linear growth directly through the $E/B$ term, the vertical drifts also affect the growth rate indirectly through the $g/\nu$_in_ term. We propose that the relatively weak background post-midnight vertical drifts that have been often observed during certain conditions (e.g., June solstice) can be weakened even more and reversed due to effects of disturbance upward drifts. In this case, observations indicate that even a modest geomagnetic storm can produce upward drifts at the magnetic equator and create conditions favoring the development of the GRT instability in the post-midnight sector.

Perhaps most importantly, the assessment of the local and non-local generation of isolated post-midnight ESF events revealed very similar occurrence rates for all geomagnetic, seasonal, and solar flux conditions. This result from long-term ESF echo analysis shows that isolated post-midnight ESF measured directly above the JRO in any season under any solar flux and geomagnetic conditions has an approximately 50/50 chance of having been generated non-locally or locally. This finding may aid understanding as to why efforts to forecast ESF with data-driven numerical models (e.g., Hysell et al. 2014) sometimes are not well-suited to explain late nighttime ESF occurrence over Jicamarca. These ESF events could have been generated to the west of Jicamarca where conditions (e.g., drifts) differ from those at the site and contribute more favorably to the ESF development. The ESF event would then drift zonally into the Jicamarca FOV, being observed later at night.

## Data Availability

The AMISR-14 RTI maps used in this study are available at the JRO database (https://www.igp.gob.pe/observatorios/radio-observatorio-jicamarca/database/dataset/?q=amisr&sort=dataset_end_date+desc – Last accessed on 15 September 2025). The MP ISR data used in this study are available in the Madrigal online database (https://www.igp.gob.pe/observatorios/radio-observatorio-jicamarca/madrigal/index.html – Last accessed on 15 September 2025). The ionosonde data used in this study are available at the JRO database (https://lisn.igp.gob.pe/database/project/ionosonde – Last accessed on 15 September 2025). The Interplanetary magnetic field z-component parameter and Sym-H and auroral electrojet indices were obtained from NASA/GSFC’s Space Physics Data Facility’s OMNIWeb service (https://omniweb.gsfc.nasa.gov/). The Planetary K and F10.7 solar radio flux indices were provided by the GFZ Helmholtz Centre for Geosciences (https://kp.gfz.de/en/).
